# Supplementary material for: Patient-derived cancer organoid tracking with wide-field one-photon redox imaging to assess treatment response
Source: J Biomed Opt. 2021 Mar 22;26(3):036005. doi: 10.1117/1.JBO.26.3.036005 (PMC7983069; doi:10.1117/1.JBO.26.3.036005)
Supplement: Supplementary file 1 [file JBO_026_036005_SD001.pdf]

Supplemental Material

Supplemental Table 1 – Definition of Quantitative Variables

Variable Name	Variable Type	Definition (units)
**Organoid Area**	Morphological: Size	Number of pixels of segmented organoid multiplied by the squared pixel size (mm^2).
**Major Axis Length**	Morphological: Size	Length of major axis of ellipse that fits the segmented organoid (i.e., normalized second image moment) multiplied by the pixel size (mm).
**Minor Axis Length**	Morphological: Size	Length of minor axis of ellipse that fits the segmented organoid (i.e., normalized second image moment) multiplied by the pixel size (mm).
**Eccentricity**	Morphological: Shape	The ratio of the distance between the foci of the ellipse and its major axis length. An ellipse with eccentricity of 0 is a circle. An ellipse with eccentricity of 1 is a line.
**Circularity**	Morphological: Shape	$(4\pi * Organoid\ Area)/(Organoid\ Perimeter^2)$. For perfect circles, the value is 1.
**Convex Area**	Morphological: Size	Number of pixels in the convex hull containing the organoid multiplied by the squared pixel size (mm^2).
**Maximum Feret's Diameter**	Morphological: Size	Maximum distance between two antipodal vertices of the convex hull (i.e., caliper diameter) multiplied by the pixel size (mm).
**Minimum Feret's Diameter**	Morphological: Size	Minimum distance between two antipodal vertices of the convex hull (i.e., caliper diameter) multiplied by the pixel size (mm).
**Equivalent Diameter**	Morphological: Shape	Diameter of a circle with the same area as the organoid defined as $(4 * Area)/(\pi)$ multiplied by the pixel size (mm).
**Solidity**	Morphological: Shape	Proportion of the pixels in the convex hull that are also in the organoid defined as $(Area)/(Convex\ Area)$.
**Extent**	Morphological: Shape	Ratio of pixels in the organoid to pixels in the total bounding box defined as $(Area)/(Bounding\ Box\ Area)$.
**Organoid Perimeter**	Morphological: Size	Distance around the boundary of the organoid multiplied by the pixel size (mm).
**Optical Redox Ratio Mean**	Metabolic	Mean value of all redox ratio pixels in the organoid.
**Optical Redox Ratio Minimum**	Metabolic	Minimum value of all redox ratio pixels in the organoid.
**Optical Redox Ratio Maximum**	Metabolic	Maximum value of all redox ratio pixels in the organoid.
**Optical Redox Ratio Standard Deviation**	Metabolic	Standard deviation of all redox ratio pixels in the organoid.
**NAD(P)H Intensity Mean**	Metabolic	Mean value of all NAD(P)H pixels in the organoid.
**NAD(P)H Intensity Minimum**	Metabolic	Minimum value of all NAD(P)H pixels in the organoid.
**NAD(P)H Intensity Maximum**	Metabolic	Maximum value of all NAD(P)H pixels in the organoid.
**NAD(P)H Intensity Standard Deviation**	Metabolic	Standard deviation of all NAD(P)H pixels in the organoid.
**FAD Intensity Mean**	Metabolic	Mean value of all FAD pixels in the organoid.
**FAD Intensity Minimum**	Metabolic	Minimum value of all FAD pixels in the organoid.
**FAD Intensity Maximum**	Metabolic	Maximum value of all FAD pixels in the organoid.
**FAD Intensity Standard Deviation**	Metabolic	Standard deviation of all FAD pixels in the organoid.

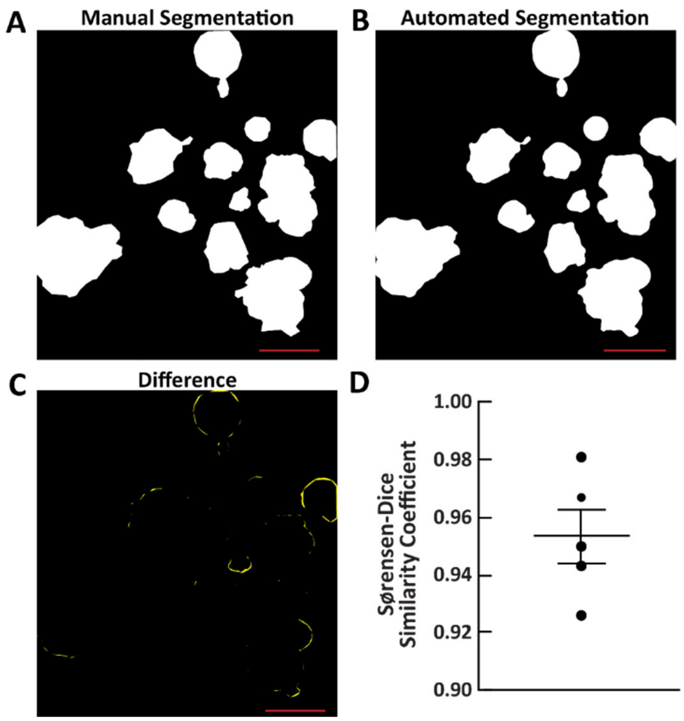

Supplemental Figure 1. Validation of the Segmentation Pipeline. The segmentation pipeline was validated by comparing the results of the automated pipeline to manual segmentation. (A) A representative image segmented manually in ImageJ. (B) The corresponding image after automated segmentation. (C) The difference between the automated and manual segmented images. (D) The Sørenson-Dice similarity coefficient for n = 5 image pairs plotted here as individual data points along with the mean and standard error. Scale bar: 500µm.

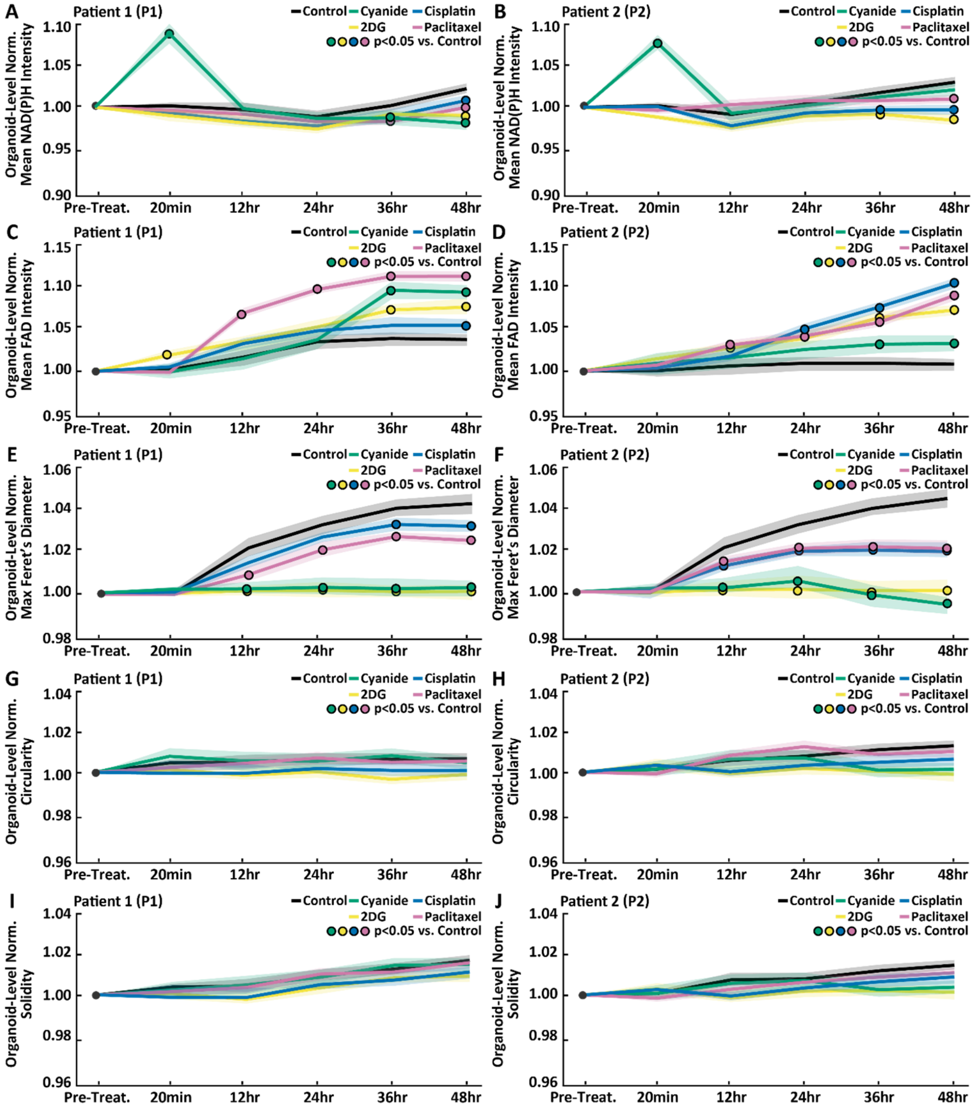

Supplemental Figure 2. Time Series of Treatment-induced Changes in Additional Redox Imaging Variables. A subset of the redox imaging variables is shown here. (A,B) Pre-treatment-normalized mean NAD(P)H intensity time series for all treatment groups for P1 and P2, respectively. (C,D) Pre-treatment-normalized mean FAD intensity time series for all treatment groups for P1 and P2, respectively. (E,F) Pre-treatment-normalized max Feret's diameter time series for all treatment groups for P1 and P2, respectively. (G,H) Pre-treatment-normalized circularity time series for all treatment groups for P1 and P2, respectively. (I,J) Pre-treatment-normalized solidity time series for all treatment groups for P1 and P2, respectively. Data plotted is mean ± standard error of the mean. Significant differences between a treatment and control is indicated with a colored circle for $p < 0.05$. Table 1 shows the number of organoids in each group and for each patient.

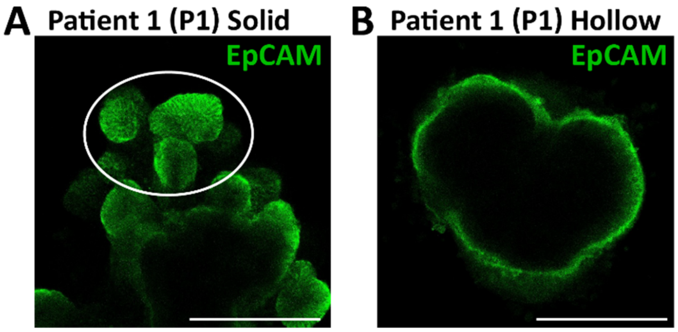

Supplemental Figure 3. Confocal Microscopy of P1 Patient-Derived Cancer Organoids. Confocal microscopy of patient-derived cancer organoids stained for epithelial cell adhesion molecular (EpCAM) illustrates the difference in the internal structure of P1 Solid and P1 Hollow patient-derived cancer organoids. (A) Confocal image of a P1 Solid patient-derived cancer organoid. The ellipse shows a region of densely organized cells characteristic of this phenotype. (B) Confocal image of a P1 Hollow patient-derived cancer organoid. Cells in these organoids are organized into a hollow shell. Scale bar: 100μm

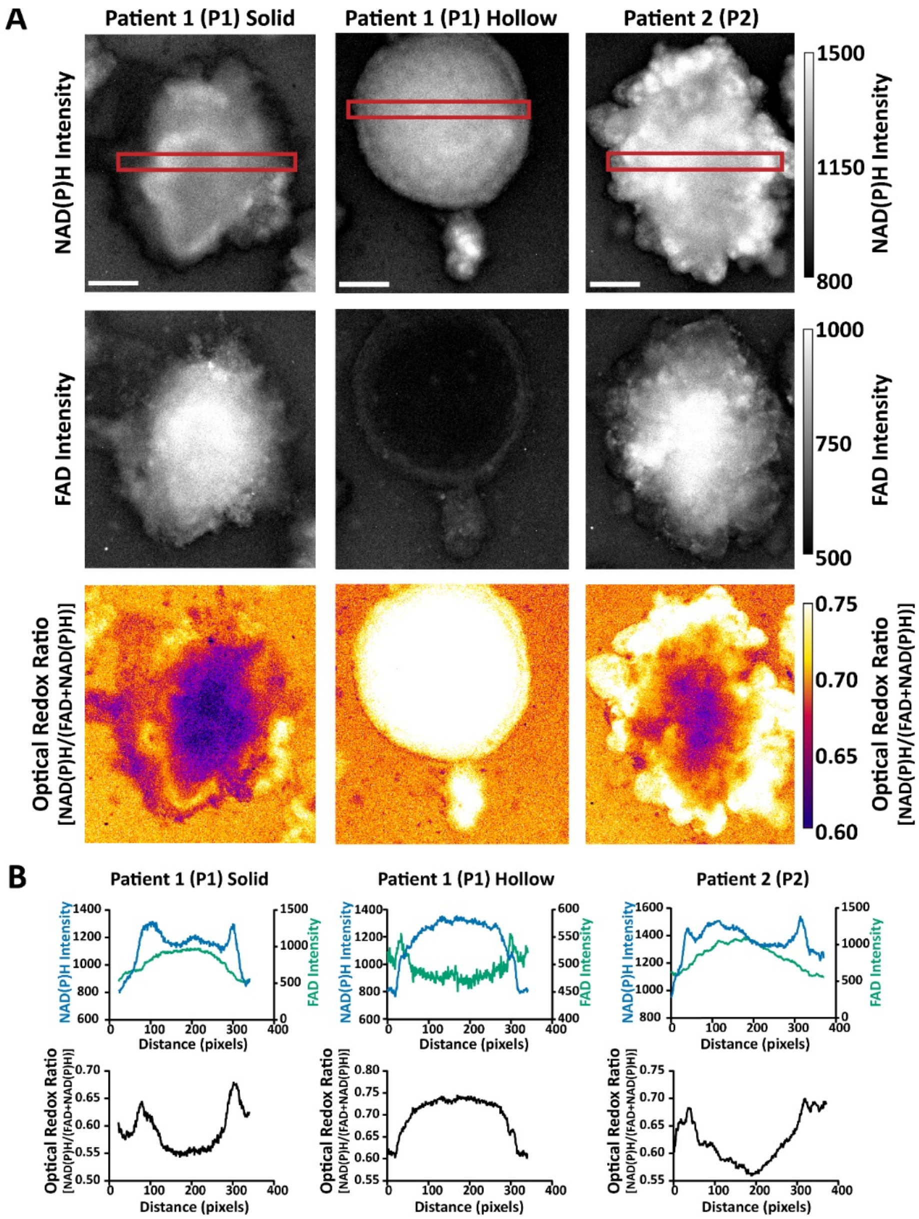

Supplemental Figure 4. Autofluorescence of Patient-Derived Cancer Organoid Sub-Types. Qualitative and quantitative data to support observations of the autofluorescence distribution of each organoid sub-type. (A) Representative NAD(P)H intensity, FAD intensity, and optical redox ratio [NAD(P)H/(FAD+NAD(P)H)] images of each of the patient-derived cancer organoid sub-types, including Patient 1 Solid (left), Patient 1 Hollow (middle), and Patient 2 (right). The red boxes indicate the region used to derive the representative line profiles of the NAD(P)H, FAD, and optical redox ratio across the organoids. (B) Representative line profiles of the NAD(P)H intensity (top, blue), FAD intensity (top, red), and optical redox ratio (bottom) for each patient-derived cancer organoid sub-type, including Patient 1 Solid (left), Patient 1 Hollow (middle), and Patient 2 (right). Scale bar: 100μm.
